# Supplementary material for: Content-rich biological network constructed by mining PubMed abstracts
Source: BMC Bioinformatics. 2004 Oct 8;5:147. doi: 10.1186/1471-2105-5-147 (PMC528731; doi:10.1186/1471-2105-5-147)
Supplement: Additional File 2 — The original results of the above study (non-essential files are deleted to keep the file size under the limit set by BMC bioinformatics). [file 1471-2105-5-147-S2.bz2 › chilibotAdditionalFile2/dip05/416ID10504300E10082/html/BMP6_ACVR2B.html]

 


 **BMP6** and **ACVR2B** 
  
Found 2 abstracts in PubMed, retrieved 2.  
 

 What does Google say? 
 PDF only 
| .edu only 

---

- Dev Dyn, 2003   **Expression and misexpression of members of the FGF and TGFbeta families of growth factors in the developing mouse pancreas.** .
  We have performed a high capacity, semiquantitative, reverse transcriptase polymerase chain reaction screen for expression of fibroblast growth factor FGF and transforming growth factor beta TGFbeta family genes as well as their cognate receptors.
  By using cDNA prepared from embryonic day 12 to postnatal day 0 embryonic mouse pancreas, we have identified several factors potentially involved in the development of the endocrine pancreas.
  We find high level early expression of TGFbeta 1 and 2, and constitutive expression of TGFbeta 3 and their receptors.
  Of the Inhibin Activin members, we found exclusively Inhibin alpha and Activin betaB to be expressed, and the BMP family was represented by BMP4, BMP5, and BMP7.
  The predominant forms of the BMP and Activin type II receptors were ActR IIB **[ACVR2B]** and BMPR II and of the type I receptors, BMPR 1A and 1B were the highest expressed.
  FGF1, FGF7, FGF9, FGF10, FGF11, and FGF18 were also expressed in the pancreas at varying time points and levels, as well as FGF receptor forms FGFR1b, FGFR1c, FGFR2b, FGFR2c, FGFR3b, and FGFR4.
  To gain insight into the biological function, we misexpressed members of these families in the pancreas by using the early pancreas promoter Pdx1.
  Misexpression of FGF4 results in disruption of the pancreas morphology with epithelial structures interspersed in stroma tissue.
  The endocrine compartment was reduced to scattered single cells, and the exocrine consisted of unbranched ductal epithelia with acinar structures budding off.
  In contrast, misexpression of BMP 6 **[BMP6]** resulted in complete agenesis of the pancreas and reduced the size of the stomach and spleen dramatically and caused fusion of the liver and duodenum.
  Developmental Dynamics 226 663 67 003.

  - Kidney Int, 2002   **BMP 7 regulates chemokine, cytokine, and hemodynamic gene expression in proximal tubule cells.** .
    BACKGROUND Proximal tubule epithelial cells PTEC play a central role in the response of the kidney to insult by virtue of their production of chemokines and cytokines that signal an inflammatory response.
    Bone morphogenic protein 7 BMP 7 OP 1 , a member of the transforming growth factor beta TGF beta superfamily, has previously been demonstrated to reduce macrophage infiltration and tissue damage in animal models of acute and chronic renal failure.
    The present study was designed to define the molecular mechanism of BMP 7 action in human PTEC.
    METHODS Expression of BMP 7 in the adult mouse kidney was determined indirectly through X gal staining of heterozygous BMP 7 lacZ mice in combination with cell type specific markers.
    Primary human PTEC were cultured in the presence of the pro inflammatory cytokine, tumor necrosis factor alpha TNF alpha , with and without BMP 7.
    RNA isolated from these two populations was then used to identify differentially regulated genes via gene array analysis.
    Modulation of potential target genes was subsequently confirmed through ELISA and or quantitative PCR.
    RESULTS Expression from the BMP 7 lacZ transgene was detected in the collecting duct, thick ascending limb, distal convoluted tubule, and podocytes within glomeruli.
    No expression was detected within PTEC.
    these cells were found to express mRNA for BMP receptors including, ActR I, BMPR IA, ActR II, ActR IIB **[ACVR2B]**, and BMPR II.
    BMP 7 significantly reduced TNF alpha stimulated increases in mRNA for the pro inflammatory genes, interleukin 6 IL 6 and interleukin 1beta IL 1beta , and the chemoattractants monocyte chemotactic protein 1 MCP 1 and interleukin 8 IL 8 in primary human PTEC.
    In addition, BMP 7 also reduced the expression of mRNA for endothelin 2 ET 2 , a vasoconstrictor, and increased the expression of mRNA for heme oxygenase 1 HO 1 , a vasodilator, although the latter was not statistically significant.
    In experiments designed to examine MCP 1 and IL 6 protein levels in response to additional TGF beta superfamily members, TGF beta1 was unable to mimic the effects of BMP 7 in reducing IL 6 production.
    However, the closely related BMP 6 **[BMP6]** exhibited similar properties to those of BMP 7.
    Each of the factors reduced MCP 1 expression.
    CONCLUSIONS BMP 7 represses the basal and TNF alpha stimulated expression of the pro inflammatory cytokines IL 6 and IL 1beta, the chemokines MCP 1 and IL 8, and the vasoconstrictor ET 2 in PTEC.
    This data are consistent with the in vivo observations that BMP 7 administration in a model of chronic and acute renal failure results in a reduction in the infiltration of macrophages in the renal interstitium.
    Taken together, these observations suggest that BMP 7 may be a novel therapeutic agent for kidney disorders involving inflammation and ischemic damage of PTEC.
